# Intense near-infrared-II luminescence from NaCeF_4_:Er/Yb nanoprobes for *in vitro* bioassay and *in vivo* bioimaging†
†Electronic supplementary information (ESI) available. See DOI: 10.1039/c8sc00927a


**DOI:** 10.1039/c8sc00927a

**Published:** 2018-05-01

**Authors:** Xialian Lei, Renfu Li, Datao Tu, Xiaoying Shang, Yan Liu, Wenwu You, Caixia Sun, Fan Zhang, Xueyuan Chen

**Affiliations:** a CAS Key Laboratory of Design and Assembly of Functional Nanostructures , Fujian Key Laboratory of Nanomaterials , Fujian Institute of Research on the Structure of Matter , Chinese Academy of Sciences , Fuzhou , Fujian 350002 , China . Email: dttu@fjirsm.ac.cn ; Email: xchen@fjirsm.ac.cn ; Fax: +86 591 63179421; b College of Materials Science and Engineering , Fujian Normal University , Fuzhou , Fujian 350007 , China; c Department of Chemistry , State Key Laboratory of Molecular Engineering of Polymers , Collaborative Innovation Center of Chemistry for Energy Materials , Fudan University , Shanghai 200433 , China

## Abstract

Near-infrared (NIR) II luminescence between 1000 and 1700 nm has attracted reviving interest for biosensing due to its unique advantages such as deep-tissue penetration and high spatial resolution. Traditional NIR-II probes such as organic fluorophores usually suffer from poor photostability and potential long-term toxicity. Herein, we report the controlled synthesis of monodisperse NaCeF_4_:Er/Yb nanocrystals (NCs) that exhibit intense NIR-II emission upon excitation at 980 nm. Ce^3+^ in the host lattice was found to enhance the luminescence of Er^3+^ at 1530 nm with a maximum NIR-II quantum yield of 32.8%, which is the highest among Er^3+^-activated nanoprobes. Particularly, by utilizing the intense NIR-II emission of NaCeF_4_:Er/Yb NCs, we demonstrated their application as sensitive homogeneous bioprobes to detect uric acid with the limit of detection down to 25.6 nM. Furthermore, the probe was detectable in tissues at depths of up to 10 mm, which enabled *in vivo* imaging of mouse organs and hindlimbs with high resolution, thus revealing the great potential of these NaCeF_4_:Er/Yb nanoprobes in deep-tissue diagnosis.

## Abstract 2

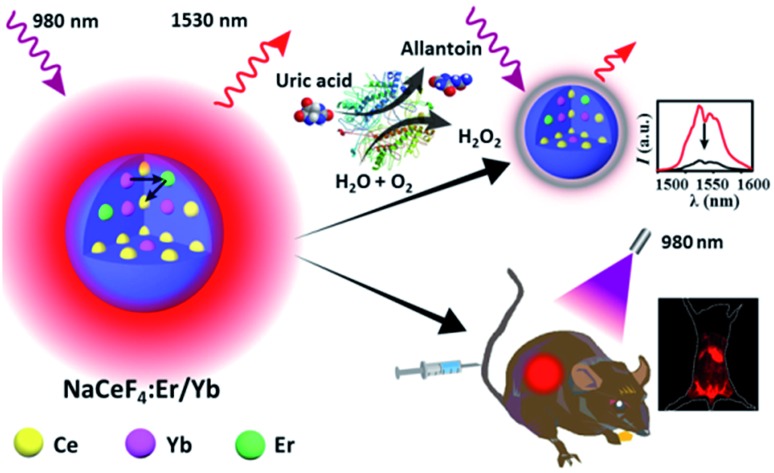
We report the controlled synthesis of monodisperse NaCeF_4_:Er/Yb nanoprobes that exhibit intense NIR-II emission for *in vitro* bioassay and *in vivo* bioimaging.

## Introduction

Luminescent biolabeling is a powerful technique that employs optical probes for detecting biomolecular concentration or visualizing biological events.^1^–^5^ In order to avoid autofluorescence and improve the signal-to-noise (S/N) ratio, several luminescent probes have emerged based on unique optical properties such as long-lived downshifting (DS) luminescence or near-infrared (NIR)-triggered upconversion (UC) luminescence.^6^–^10^ Generally, the emission lights for these probes are located below 1000 nm, which is not optimal in bioapplications since the photon scattering may limit the tissue penetration depth. To solve this problem, luminescent materials exhibiting NIR-II emission (1000–1700 nm) in the second biological window have recently been proposed as an excellent class of probes that can significantly reduce light scattering and increase the probing depth in bioapplications.^11^–^13^

In the past few years, continuous efforts have been dedicated to developing NIR-II probes including organic fluorophores, carbon nanotubes, and semiconductor quantum dots (QDs).^14^–^17^ However, the use of these bioprobes has several limitations. For example, organic fluorophores commonly possess poor photostability and are susceptible to photobleaching. The applicability of QDs is compromised by photoblinking and high toxicity of heavy metal elements (*e.g.*, cadmium and selenium). Moreover, both organic fluorophores and QDs may induce high background noise owing to a small Stokes shift, which decreases the detection sensitivity for bioassays. These concerns fuel high demand for a new generation of luminescent probes to circumvent the limitations of traditional ones.^18^

Lanthanide (Ln^3+^)-doped nanocrystals (NCs), as another kind of promising luminescent probe, have received growing attention due to their tunable emissions from different Ln^3+^ activators.^19^–^22^ Compared with organic fluorophores and QDs, Ln^3+^-doped NCs feature long luminescence lifetime, high photostability, low toxicity and sharp f–f emission peaks. Thus, they are widely applied for *in vitro* bioassays and *in vivo* bioimaging.^23^,^24^ Nevertheless, most previous research studies focused on the exploration of UC nanoprobes with emission light in the UV or visible range,^25^,^26^ which may restrict the tissue penetration depth. Several Ln^3+^ ion (*e.g.*, Pr^3+^, Nd^3+^, Sm^3+^, Dy^3+^, Ho^3+^, Er^3+^, Tm^3+^ and Yb^3+^) doped NCs have been reported to emit NIR-II light.^27^ However, the NIR-II quantum yields for most of these Ln^3+^-based NCs are still low for practical application. To meet the requirement of sensitive bioassays, it is urgent to develop Ln^3+^-doped NCs with highly efficient emission in the NIR-II region.

**Scheme 1:**
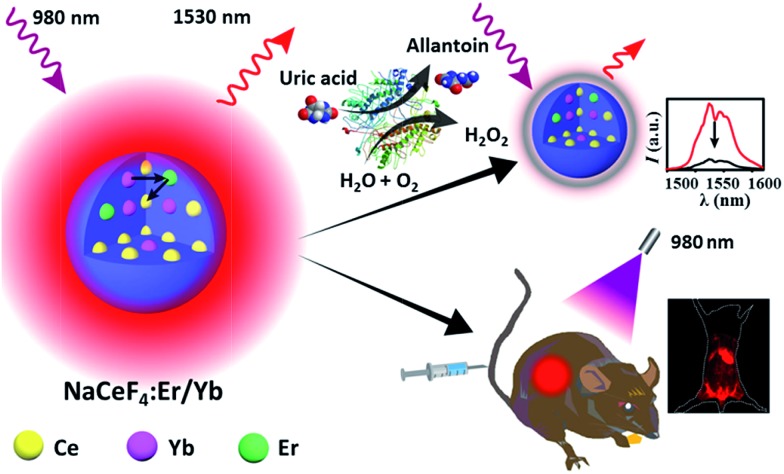
Schematic illustration showing *in vitro* bioassays and *in vivo* bioimaging based on NaCeF_4_:Er/Yb nanoprobes.

In this regard, we herein report the synthesis of monodisperse and size controllable Er^3+^/Yb^3+^-doped hexagonal NaCeF_4_ core-only and core/shell NCs that exhibit intense NIR-II emission upon 980 nm excitation, by virtue of the efficient Yb^3+^–Er^3+^–Ce^3+^ energy transfer. The maximum NIR-II quantum yield for the NaCeF_4_:Er/Yb NCs is determined to be 32.8%, which is ∼17.5 times higher than that of the widely reported NaYF_4_:Er/Yb NCs. After surface modification, these NaCeF_4_:Er/Yb nanoprobes can be applied for sensitive and selective detection of uric acid (UA) in human serum through a simple mix-and-measure type assay, with the limit of detection (LOD) down to 25.6 nM. Moreover, the tissue penetration depth of NIR-II emission from the proposed probe is found to be higher than that of the green UC emission of NaYF_4_:Er/Yb NCs of similar particle sizes under otherwise identical conditions. After tail vein injection of hydrophilic NaCeF_4_:Er/Yb@NaCeF_4_ NCs into nude mice, the biodistribution of the nanoprobes is clearly monitored for 24 h using an *in vivo* bioimaging system (Scheme 1).

## Results and discussion

**Fig. 1:**
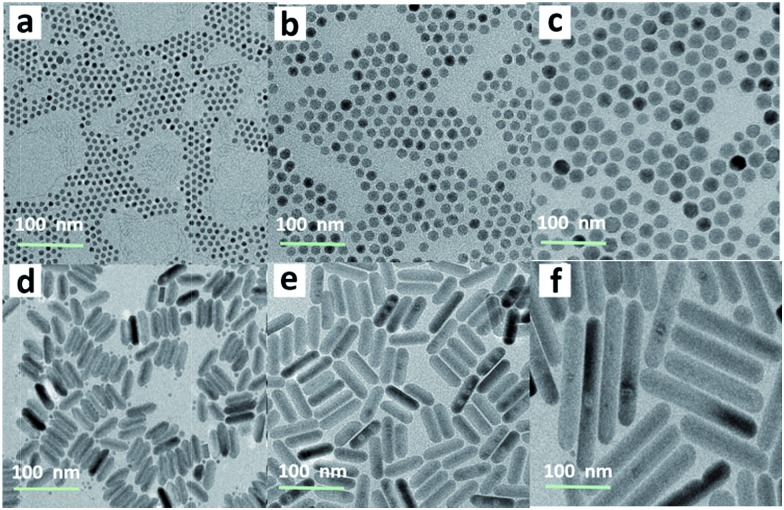
NaCeF_4_:Er/Yb NCs synthesized *via* a high-temperature co-precipitation method at 320 °C for (a) 20 min, (b) 25 min, (c) 30 min, (d) 45 min, (e) 60 min and (f) 90 min, respectively. The size of NaCeF_4_:Er/Yb NCs can be estimated by randomly analyzing 200 particles.

Hydrophobic and monodisperse NaCeF_4_:Er/Yb NCs were synthesized *via* a facile high-temperature co-precipitation method.^28^ The X-ray diffraction (XRD) patterns of the as-prepared NCs can be indexed to pure hexagonal NaCeF_4_ (JCPDS no. 75-1924), and no traces of other phases or impurities were detected (ESI Fig. S1†). Energy-dispersive X-ray (EDX) spectroscopy confirms the successful doping of Er^3+^/Yb^3+^ ions into the NaCeF_4_ host (ESI Fig. S1†). By changing the reaction time at 320 °C, the sizes and morphologies of these NCs can be finely tailored. Specifically, longer reaction time resulted in larger particles. As shown in Fig. 1, when the reaction time increased from 20 to 30 min, the size of the obtained NaCeF_4_:Er/Yb NCs increased markedly from 7.1 ± 0.5 to 25.2 ± 2.7 nm (Fig. 1a–c). With further increasing the reaction time to 45 min, the coexistence of small nanospheres and large nanorods was observed (Fig. 1d), which may be attributed to an Ostwald-ripening process where small particles dissolved and big nanorods grew simultaneously. After heating for 60 or 90 min, the small nanospheres completely transformed into larger nanorods with lengths of 103.3 ± 10.9 and 200.6 ± 16.5 nm (Fig. 1e–f), respectively.

Besides the core-only NCs, core/shell NCs were also synthesized through epitaxial growth of inert NaCeF_4_ shells on the core-only NCs (7.1 ± 0.5 nm). These core/shell NCs, with an average size of 18.1 ± 1.9 nm, can be well dispersed in nonpolar organic solvents such as cyclohexane to form a stable transparent colloidal solution (ESI Fig. S2†). The high-resolution TEM (HRTEM) image shows a clearly observed *d*-spacing of 0.308 nm, which is in good agreement with the lattice spacing in the (0111) planes of hexagonal NaCeF_4_, indicative of the high crystallinity of the as-prepared NCs.

**Fig. 2:**
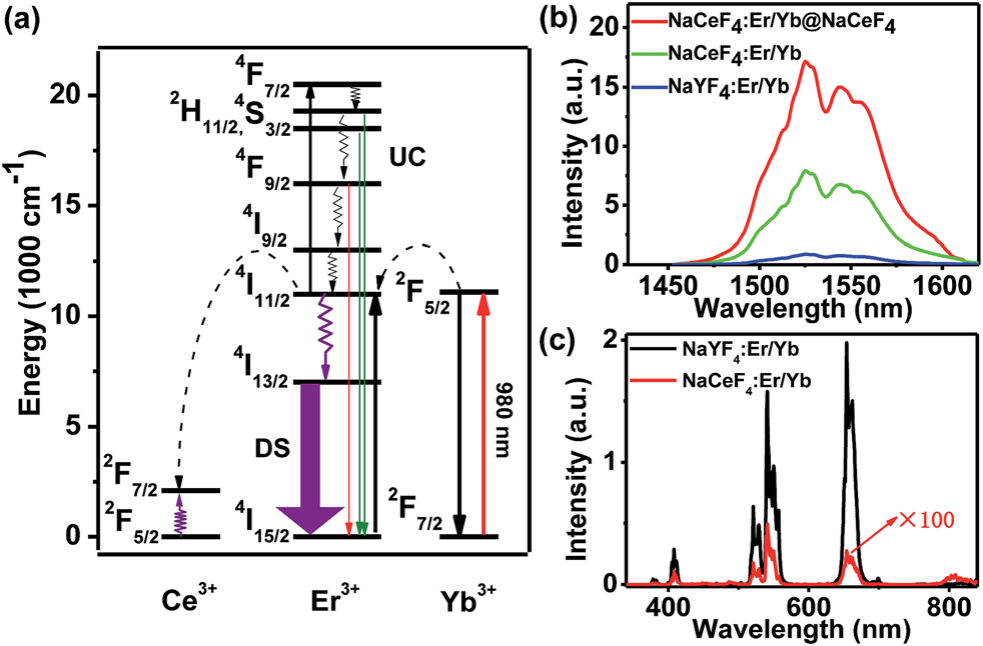
(a) Schematic mechanism for the energy-transfer process in the UC and DS emissions of Er^3+^ ions. The curly and straight arrows denote multi-phonon nonradiative transitions and radiative transitions, respectively. (b) NIR-II emission spectra of NaYF_4_:Er/Yb, NaCeF_4_:Er/Yb and NaCeF_4_:Er/Yb@NaCeF_4_ NCs, respectively, upon excitation at 980 nm. (c) UC emission spectra of NaYF_4_:Er/Yb and NaCeF_4_:Er/Yb NCs, respectively, upon excitation at 980 nm.

Currently, Er^3+^/Yb^3+^-doped fluorides (*e.g.*, NaYF_4_) with low phonon energy are frequently reported as UC materials. For the typical UC emission process, the Yb^3+^ ion is usually used as the sensitizer to harvest 980 nm photons. An Er^3+^ ion is then excited to its excited states *via* two or more successive energy transfers from Yb^3+^ ions in close proximity, followed by radiative relaxation, resulting in UC emission of a higher-energy photon (Fig. 2a).^29^ Nevertheless, the energy gap between the ^2^F_5/2_ and ^2^F_7/2_ levels of Ce^3+^ (∼2300 cm^–1^) is close to that of the ^4^I_11/2_–^4^I_13/2_ energy gap (∼3700 cm^–1^) of Er^3+^. Therefore, for NaCeF_4_:Er/Yb NCs, the ^4^I_13/2_ level of Er^3+^ is significantly populated through the efficient phonon-assisted nonradiative relaxation from the ^4^I_11/2_ level facilitated by Ce^3+^ ions.^30^ Upon excitation at 980 nm, intense DS emissions centered at ∼1530 nm that are ascribed to the ^4^I_13/2_ → ^4^I_15/2_ transition of Er^3+^ were detected for all the synthesized Er^3+^/Yb^3+^ co-doped NaCeF_4_ NCs (ESI Fig. S3 and S4†). With the size increasing from 7.1 nm to 200.6 nm, the NIR-II emission intensity increased by 4.1 times, and the effective PL lifetime of ^4^I_13/2_ was found to increase from 1.53 to 5.60 ms (ESI Fig. S5†).

The NIR-II absolute quantum yield (QY), defined as the ratio of the number of emitted photons to the number of absorbed photons, was determined to be as high as 32.8% for NaCeF_4_:Er/Yb NCs with a size of 200.6 nm upon excitation by a 980 nm laser with a power density of ∼100 W cm^–2^ (ESI Fig. S3†). Meanwhile, impressive 3.6-fold and 13.6-fold enhancements of NIR-II emission at ∼1530 nm for NaCeF_4_:Er/Yb core-only and NaCeF_4_:Er/Yb@NaCeF_4_ core/shell NCs were observed relative to that of NaYF_4_:Er/Yb NCs (20.1 ± 1.8 nm, ESI Fig. S6†), upon excitation at 980 nm (Fig. 2b). In sharp contrast, the visible UC emissions for Er^3+^ were negligibly weak in NaCeF_4_:Er/Yb NCs, due to the effective depopulation of ^2^H_11/2_, ^4^S_3/2_, and ^4^F_9/2_ levels in the presence of Ce^3+^ ions (Fig. 2c). The NIR-II absolute QYs were determined to be 1.9%, 5.6% and 19.5% for NaYF_4_:Er/Yb, NaCeF_4_:Er/Yb core-only, and NaCeF_4_:Er/Yb@NaCeF_4_ core/shell NCs, respectively.

To make the OA-capped NaCeF_4_:Er/Yb NCs hydrophilic for bioapplications, we removed the surface ligands through an acid treatment.^31^ The successful synthesis of ligand-free NaCeF_4_:Er/Yb NCs was verified by TGA, FTIR spectra and zeta-potential analyses (ESI Fig. S7–S9†). More importantly, the ligand-free NCs preserved the intense NIR-II emission from the OA-capped NCs with essentially unchanged intensity. The *ζ* potential of ligand-free NCs in aqueous solution was measured to be 21.9 ± 0.9 mV (ESI Fig. S9†) due to the existence of positively charged Ln^3+^ ions (*i.e.*, Er^3+^, Yb^3+^ and Ce^3+^) on the surface of ligand-free NCs, which endows these NCs with excellent dispersibility in aqueous solutions.

**Fig. 3:**
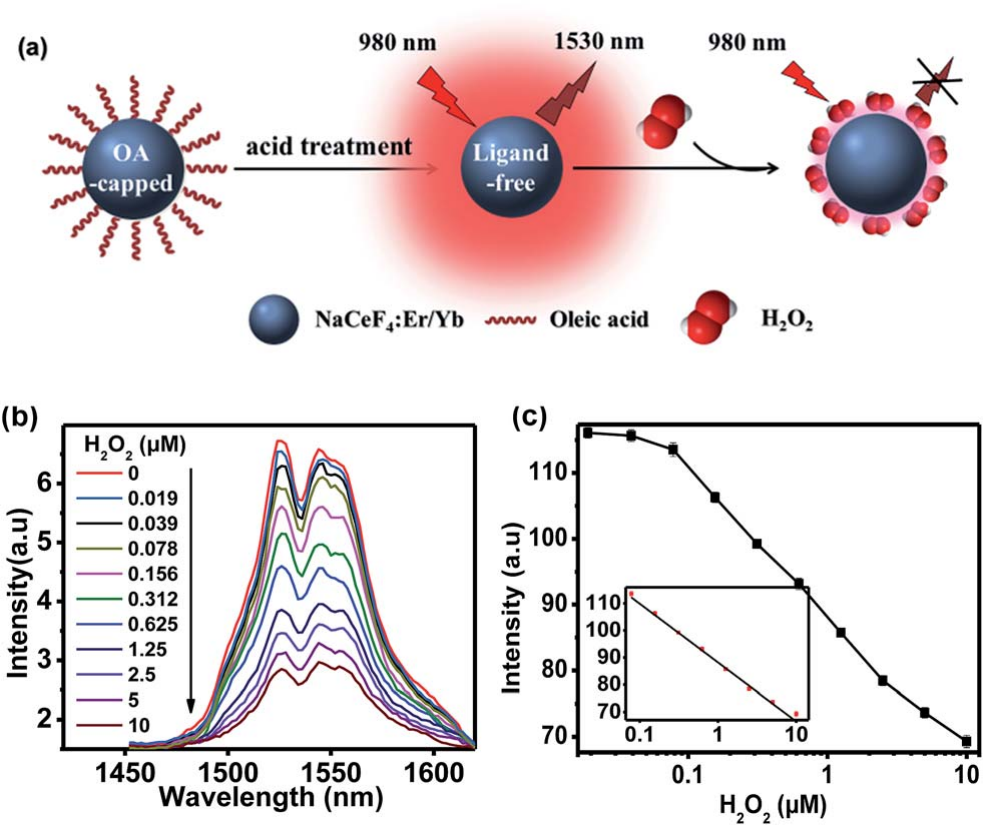
(a) Process and principle of homogeneous assay of H_2_O_2_ by employing NaCeF4:Er/Yb as the probe. (b) NIR-II emission spectra of ligand-free NaCeF_4_:Er/Yb NCs after the addition of different concentrations of H_2_O_2_ upon excitation at 980 nm. (c) Calibration curve for the H_2_O_2_ assay. The inset shows the linear range (0.078–10 μM) of the calibration curve.

Since Ce^3+^ ions in the host matrix were exposed on the surface of ligand-free NCs after the acid treatment, H_2_O_2_ can directly oxidize Ce^3+^ to Ce^4+^ through redox reaction,^32^ resulting in the quenching of NIR-II emission of Er^3+^ upon 980 nm excitation. Benefiting from such a redox reaction, NaCeF_4_:Er/Yb NCs can be explored as an effective bioprobe for the detection of H_2_O_2_ or H_2_O_2_-generated biomolecules (Fig. 3a). In order to investigate the quenching effect of H_2_O_2_ on the NIR-II emission of NaCeF_4_:Er/Yb NCs, the spectral response of ligand-free NaCeF_4_:Er/Yb NCs with a size of 25.2 ± 2.7 nm (0.5 mg mL^–1^) upon addition of different amounts of H_2_O_2_ (0–10 μM) was measured upon 980 nm excitation (Fig. 3b). The integrated DSL intensity of NaCeF_4_:Er/Yb decreased gradually with increasing concentration of H_2_O_2_, due to the redox reaction between the H_2_O_2_ and Ce^3+^ ions. As a result, the concentration of H_2_O_2_ can be quantified by the NIR-II emission intensity of NaCeF_4_:Er/Yb NCs (Fig. 3c). In the control experiment, by utilizing NaYF_4_:Er/Yb or NaYF_4_:Er/Yb/Ce (with a Ce^3+^ content of 10 mol%) as the probe, a negligible photoluminescence (PL) quenching effect of Er^3+^ was observed upon addition of different concentrations of H_2_O_2_ (ESI Fig. S10†). The LOD, defined as the concentration that corresponds to 3 times the standard deviation above the signal measured in the blank, was determined to be 41.8 nM based on NaCeF_4_:Er/Yb nanoprobes.

**Fig. 4:**
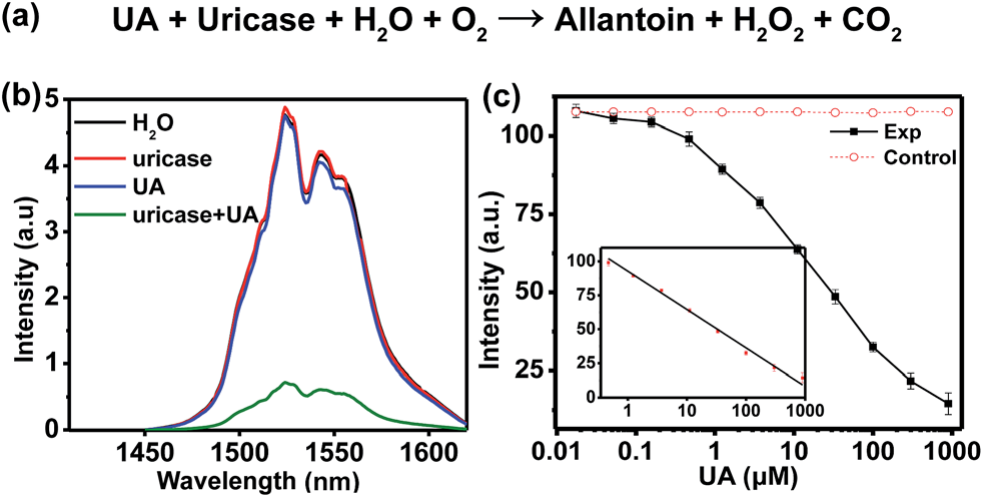
(a) Chemical equation for the generation of H_2_O_2_ through UA/uricase reaction. (b) NIR-II emission spectra of 100 μL of NaCeF_4_:Er/Yb NCs (0.5 mg mL^–1^) after the addition of 100 μL of H_2_O, uricase (0.011 U mL^–1^), UA (900 μM) and uricase (0.011 U mL^–1^) + UA (900 μM), respectively, upon excitation at 980 nm. (c) Calibration curve for the UA assay. The control experiment was carried out by replacing UA with H_2_O under otherwise identical conditions. The inset shows the linear range (0.411–900 μM) of the calibration curve.

The highly sensitive response of H_2_O_2_ allows for the detection of biomarkers such as UA which can yield H_2_O_2_ through the UA/uricase reaction (Fig. 4a). The level of UA, which is the end product of purine metabolism in the human body in human blood and urine, can be treated as an indicator for certain clinical criteria. Abnormal levels of UA may cause diseases like gout, arthritis, renal disorder, Lesch–Nyhan syndrome, *etc.*^33^,^34^ Specifically, excess UA in human blood is a risk factor in cardiovascular related diseases, while reduced UA levels (hypouricemia) have been found to be closely related to several diseases such as diabetes mellitus and AIDS.^35^ Therefore, the accurate detection of UA is of great importance in physiological survey and clinical diagnosis.

**Fig. 5:**
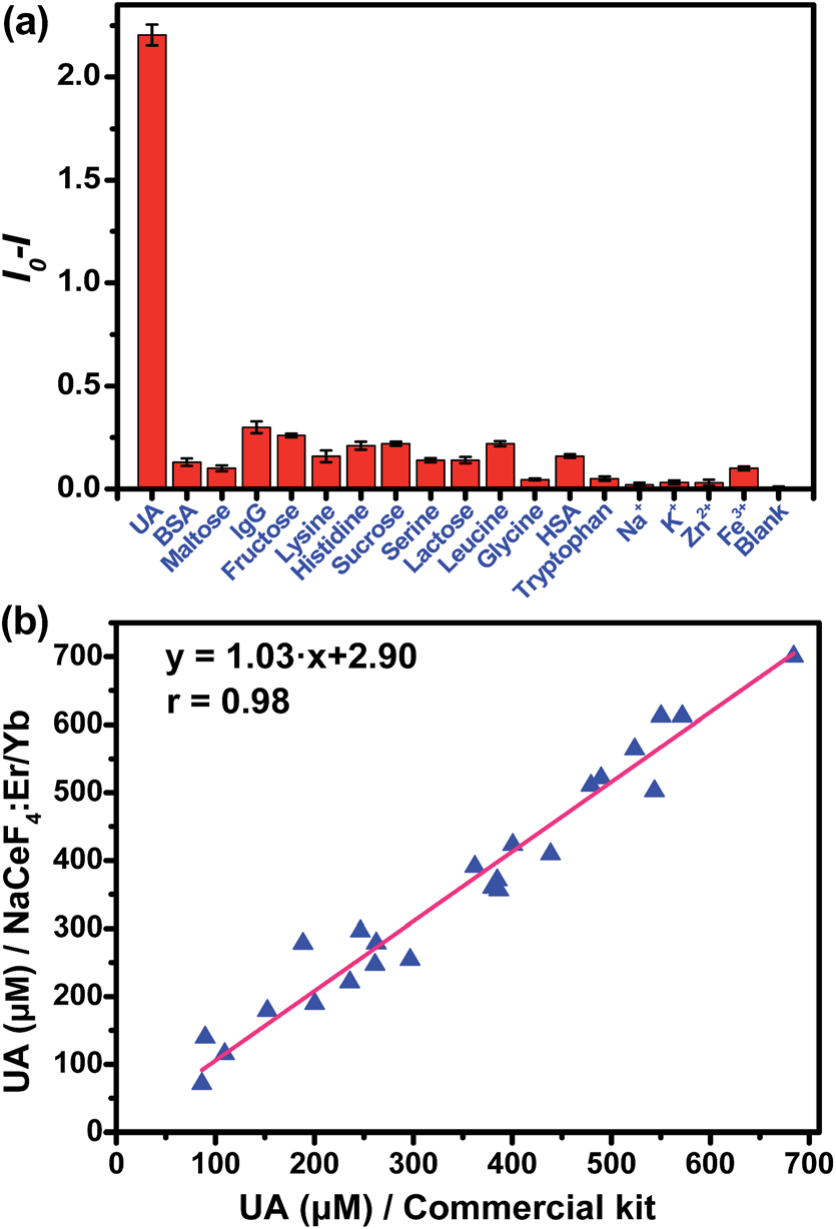
(a) Quenching effect of NIR-II signal at 1530 nm of the NCs/uricase solution under 980 nm excitation after incubation with 1 mM UA or other different analytes. Error bars represent the standard deviations of triplicate experiments. (b) Correlation between NCs-based assay and commercial kit for the detection of UA in 24 human serum samples, which were kindly provided by Fujian Provincial Cancer Hospital, Fuzhou, China. Each data point represents the mean of triplicate experiments.

In the assay system, UA or uricase alone was not able to quench the NIR-II emission of NaCeF_4_:Er/Yb nanoprobes upon 980 nm excitation, since no H_2_O_2_ was generated (Fig. 4b). However, a notable quenching in Er^3+^ emission was observed with the addition of both UA and uricase in NaCeF_4_:Er/Yb solution. Meanwhile, it was found that a time of 3 h was needed to reach equilibrium for the NIR-II emission of Er^3+^ (ESI Fig. S11†). Under the optimized conditions (0.5 mg mL^–1^ NaCeF_4_:Er/Yb and 0.011 U mL^–1^ uricase), the integrated NIR-II emission intensity of Er^3+^ decreased gradually with UA concentration from 0 to 900 μM (Fig. 4c), due to the gradual release of H_2_O_2_. The calibration curve for the UA concentration exhibits a linear dependence in the range of 0.411–900 μM. The LOD of UA assay was determined to be 25.6 nM, which is much lower than the UA level in the serum of healthy human beings (130–460 μM).^35^ In order to verify the specificity of the bioassay, we performed control experiments by replacing UA with other possible interfering biomolecules and electrolytes that may exist in serum samples, such as metal ions, proteins, and amino acids, under otherwise identical conditions. As displayed in Fig. 5a, the quenching of NIR-II emission of Er^3+^ in the control groups was negligibly small, which is in marked contrast to the significant quenching effect caused by the addition of UA. Such an exclusive PL quenching in the experiment group confirms the high specificity of the assay, thus validating the applicability of NaCeF_4_:Er/Yb nanoprobes for UA detection in complex biological matrices such as serum.

For the detection of UA in human serum samples, the NIR-II signal of the serum-based detection system exhibited a linear dependence on the UA concentration ranging from 1.234 to 900 μM (ESI Fig. S12†). To show the reliability of direct quantitation of UA in complex biological fluids by applying the NaCeF_4_:Er/Yb nanoprobes, we carried out *in vitro* detection of UA in 24 serum samples. The UA concentrations determined by NaCeF_4_:Er/Yb nanoprobes were compared with those detected based on a commercial kit. As shown in Fig. 5b and Table S1,† the UA levels determined from the NaCeF_4_:Er/Yb based assay are highly consistent with those from the commercial assay kit. The correlation coefficient between both kinds of assays was determined to be 0.98, demonstrating that the NC-based assay is as reliable as that using the commercial kit.

Moreover, we determined the recovery of three human serum samples upon addition of UA standard solutions with different concentrations. The analytical recoveries are in the range of 93.4–108.8% (Table 1). Both the coefficients of variation (CV) and recovery are within the acceptance criteria (CVs ≤ 15%, and recoveries in the range of 90–110%) set for bioanalytical method validation.^36^ These results clearly prove that the NaCeF_4_:Er/Yb nanoprobe has high reliability and practicability for UA detection in complex biological samples. Therefore, the proposed NaCeF_4_:Er/Yb nanoprobe, exhibiting background-free NIR-II emission under NIR excitation, is highly desired as a homogeneous bioassay nanoplatform for accurate detection of UA and other H_2_O_2_-generated biomarkers in clinical bioassays. Compared to previously reported UA bioassay systems, the homogeneous assay carried out employing the NaCeF_4_:Er/Yb nanoprobe is much more convenient and cost-effective, given that the assay can be performed based on a simple mixing of the test samples with uricase and the ligand-free NaCeF_4_:Er/Yb nanoprobe, and no complicated operations are involved in either nanoprobe preparation or surface modification.

**Table 1:** Assay precision and analytical recovery of UA added to three serum samples from healthy people

Added (μM)	Found (μM)	CV (%) *n* = 4	Recovery (%)
Serum 1	145.6	5.1	—
50	198.2	6.2	105.3%
100	254.4	4.3	108.8%
200	332.5	2.7	93.4%
Serum 2	208.0	3.5	—
50	259.4	7.6	102.9%
100	311.4	6.6	103.4%
200	417.8	3.9	104.9%
Serum 3	189.3	2.4	—
50	237.2	8.2	95.7%
100	287.5	5.9	98.1%
200	396.8	4.4	103.7%

**Fig. 6:**
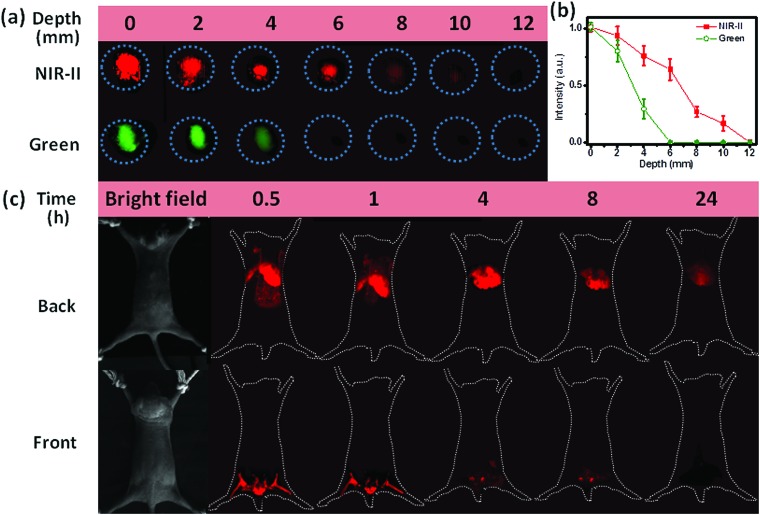
(a) Comparison of the NIR-II emission signal of NaCeF_4_:Er/Yb@NaCeF_4_ NCs and the green UC emission signal of NaYF_4_:Er/Yb NCs covered by pork muscle tissue of various thicknesses upon excitation at 980 nm with a power density of ∼0.5 W cm^–2^. (b) Integrated signal intensity from NaCeF_4_:Er/Yb@NaCeF_4_ and NaYF_4_:Er/Yb NCs (marked with a circle) at different depths. Both NIR-II and green emission intensities were normalized at 0 mm. (c) NIR-II photographs of *in vivo* front and back images of nude mice after tail vein injection of Lipo-modified NaCeF_4_:Er/Yb@NaCeF_4_ NCs at different times. All images and spectra were taken under a 980 nm laser excitation with a power density of ∼0.2 W cm^–2^. All animal procedures were performed in accordance with the Guidelines for Care and Use of Laboratory Animals of Fudan University, and the protocol was approved by the Animal Ethics Committee of Fudan University.

Another important application of NIR-II emission is the deep-tissue bioimaging. To make the as-prepared hydrophobic NCs biocompatible, we coated the surface of OA-capped NaCeF_4_:Er/Yb@NaCeF_4_ NCs with amphiphilic 1,2-distearoyl-*sn-glycero*-3-phosphoethanolamine-*N*-[carboxy-(polyethyleneglycol)-2000] (DSPE-PEG2000-COOH) phospholipids (Lipo).^37^ The resultant Lipo-modified NaCeF_4_:Er/Yb@NaCeF_4_ NCs with a hydrodynamic diameter of 22.3 ± 1.1 nm were monodisperse in water (ESI Fig. S9†). As a proof-of-concept experiment to examine the tissue penetration ability of NIR-II emission, we covered Lipo-modified NaCeF_4_:Er/Yb@NaCeF_4_ and NaYF_4_:Er/Yb NCs with pork muscle tissue of various thicknesses, which were imaged using a modified Maestro imaging system. As shown in Fig. 6a and b, the NIR-II luminescence of NaCeF_4_:Er/Yb@NaCeF_4_ NCs was detectable even at a depth of 10 mm upon excitation at 980 nm. By contrast, the green UC luminescence of NaYF_4_:Er/Yb NCs can only be observed at 4 mm beneath the tissue surface under otherwise identical conditions. The penetration depths that correspond to 50% of the original signal of NIR-II and green luminescence were determined to be ∼7 and ∼3 mm, respectively. The higher depth of penetration of NIR-II emission is due to reduced tissue scattering of light within the NIR-II window compared with that in the visible range.

Furthermore, to demonstrate their great capability for noninvasive imaging, *in vivo* bioimaging experiments were carried out based on the Lipo-modified NaCeF_4_:Er/Yb@NaCeF_4_ and NaYF_4_:Er/Yb nanoprobes *via* tail vein injection into mice with the same dosage (0.1 mg mL^–1^, 1 mL). After 30 min of blood circulation, images were taken upon excitation at 980 nm with appropriately equipped filters. Fig. 6c shows the evolution of the PL signal over 24 h arising from the injection of NaCeF_4_:Er/Yb@NaCeF_4_ nanoprobes. 0.5 h after injection, the NCs accumulated essentially in the hindlimbs, liver, spleen, and lungs, as can be monitored by the bright NIR-II signals of Er^3+^. Particularly, images of the mouse blood vessels of organs and hindlimbs can be clearly observed, which reveals the excellent spatial resolution of NIR-II bioimaging. After longer time periods of blood circulation, PL fading from hindlimbs was observed. 24 h later, all the NaCeF_4_:Er/Yb@NaCeF_4_ nanoprobes accumulated in the liver. Note that no tissue autofluorescence signal and light scattering were detected during *in vivo* imaging experiments. Considering the depth of the nude-mice organs (>3 mm), only weak visible UC emission of Er^3+^ can be observed in the control experiment by utilizing NaYF_4_:Er/Yb as the probe under otherwise identical conditions (ESI Fig. S13†). These results are well consistent with the penetration depths of NIR-II and visible lights verified in the above-mentioned *in vitro* imaging experiments with pork tissue.

## Conclusions

In summary, we have developed a highly efficient NIR-II nanoprobe based on NaCeF_4_:Er/Yb NCs. Upon 980 nm excitation, intense NIR-II emissions at 1530 nm were realized because of efficient Yb^3+^–Er^3+^–Ce^3+^ energy transfer. The maximum absolute NIR-II QY for NaCeF_4_:Er/Yb NCs has been determined to be 32.8%, which is the highest among Er^3+^-activated NIR-II nanoprobes. Significantly, the NIR-II emission can be effectively inhibited by H_2_O_2_ produced *via* the UA/uricase reaction, due to the redox reaction between the H_2_O_2_ and Ce^3+^ ions. By virtue of such H_2_O_2_-responsive luminescence, we have achieved an LOD of 25.6 nM for UA detection. The concentrations of UA in 24 human serum samples determined using NaCeF_4_:Er/Yb nanoprobes were highly consistent with those measured independently using a commercial kit, showing the assay's accuracy and reliability. More importantly, a deep tissue penetration depth and superior spatial resolution in *in vivo* imaging of mouse organs and hindlimbs have been demonstrated by employing the distinct NIR-II emission of NaCeF_4_:Er/Yb@NaCeF_4_ in comparison with the UC emission of NaYF_4_:Er/Yb. These findings reveal the great potential of NaCeF_4_:Er/Yb nanoprobes in practical *in vivo* detection of disease markers, which may open up a new route to the exploitation of Ln^3+^-doped NIR-II nanoprobes in versatile biomedical applications.

## Experimental

Detailed experimental procedures are reported in the ESI.†

## Conflicts of interest

There are no conflicts to declare.

## Supplementary Material

Supplementary information
