# Supplementary material for: Intense near-infrared-II luminescence from NaCeF4:Er/Yb nanoprobes for in vitro bioassay and in vivo bioimaging
Source: Chem Sci. 2018 May 1;9(20):4682–8. doi: 10.1039/c8sc00927a (PMC5969504; doi:10.1039/c8sc00927a)
Supplement: Supplementary file 1 [file SC-009-C8SC00927A-s001.pdf]

Electronic Supplementary Material (ESI) for Chemical Science.
This journal is © The Royal Society of Chemistry 2018

Electronic Supplementary Information (ESI) for Chemical Science

Intense Near-Infrared-II Luminescence from $NaCeF_4$:Er/Yb Nanoprobes for *in vitro* Bioassay and *in vivo* Bioimaging

Xialian Lei,[ab] Renfu Li,[a] Datao Tu,*[ab] Xiaoying Shang,[a] Yan Liu,[a] Wenwu You,[a] Caixia Sun,[c] Fan Zhang,[c] and Xueyuan Chen*[ab]

[a] CAS Key Laboratory of Design and Assembly of Functional Nanostructures, and Fujian Key Laboratory of Nanomaterials, Fujian Institute of Research on the Structure of Matter, Chinese Academy of Sciences, Fuzhou, Fujian 350002, China.

[b] College of Materials Science and Engineering, Fujian Normal University, Fuzhou, Fujian 350007, China.

[c] Department of Chemistry, State Key Laboratory of Molecular Engineering of Polymers, Collaborative Innovation Center of Chemistry for Energy Materials, Fudan University, Shanghai 200433, China.

Fax/Tel: +86-591-63179421; E-mail: dttu@fjirsm.ac.cn, xchen@fjirsm.ac.cn

I. Supplementary Materials & Methods

Chemicals and Materials: Oleic acid (OA), 1-octadecence (ODE), oleylamine (OM), uric acid (UA) and uricase were purchased from Sigma-Aldrich, China. NaOH, NaOA, $Ce(Ac)_3$, $Er(Ac)_3$, $Yb(Ac)_3$, NH_4F, cyclohexane, chloroform, hydrogen peroxide (H_2O_2) and hydrochloric acid (HCl) were purchased from Sinopharm Chemical Reagent Co., China. Ethanol and methanol were purchased from Adamas-beta Ltd. (Shanghai, China). 1,2-distearoyl-sn-glycero-3-phosphoethanolamine-N-[carboxy-(polyethyleneglycol)-2000] (DSPE-PEG2000-COOH) phospholipids (Lipo) was purchased from Shanghai Yare Biotech Inc. N-ethyl maleimide (>98%, NEM) was from J&K Scientific Ltd., Shanghai. The 96-well microplate was purchased from Thermo Fisher Scientific Inc. Commercial kit for UA was ordered from Shanghai Linc-Bio Science Co., China. All the chemical reagents were of analytical grade and used directly without any further purification. Distilled water was used throughout.

Synthesis of $NaCeF_4$:Er/Yb core-only nanocrystals (NCs): Mondisperse $NaCeF_4$:Er/Yb core-only NCs were synthesized via a high-temperature co-precipitation method. 2.5 mmol of NaOA, 0.01 mmol of $Er(Ac)_3$, 0.2 mmol of $Yb(Ac)_3$ and 0.79 mmol of $Ce(Ac)_3$ were added to a 100 mL three-neck round-bottom flask containing 5 mL of OA, 10 mL of ODE and 5 mL of OM. The resulting mixture was heated to 160 °C under N_2 flow with constant stirring for 30 min to form a clear solution, and then cooled down to room temperature (RT). 10 mL of methanol solution containing 4 mmol of NH_4F was added to the solution, which was stirred at 60 °C for 30 min to remove methanol. Afterwards, the solution was stirred at 120 °C for 20 min to remove H_2O. Finally, the resulting solution was heated to 320 °C under N_2 flow with vigorous stirring for 20-90 min, and cooled down to RT. The obtained $NaCeF_4$:Er/Yb NCs were precipitated by addition of 30 mL of ethanol, collected by centrifugation, washed with ethanol several times, and redispersed in cyclohexane.

Synthesis of $NaCeF_4$:Er/Yb@$NaCeF_4$ core/shell NCs: Briefly, 2.5 mmol of NaOA and 1 mmol of $Ce(Ac)_3$ were added to a 100 mL three-neck round-bottom flask containing 5 mL of OA, 10 mL of ODE and 5 mL of OM. The resulting mixture was heated to 160 °C under N_2 flow with constant stirring for 30 min to form a clear solution. After cooling down to 80 °C, 0.5 mmol of $NaCeF_4$:Er/Yb core-only NCs in 10 mL of cyclohexane was added and the solution was maintained at 80 °C for 30 min to remove cyclohexane. Then, 10 mL of methanol solution containing 4 mmol of NH_4F was added and stirred at 60 °C for another 30 min to remove methanol. Subsequently, the solution was stirred at 120 °C for 20 min to remove H_2O. Finally, the solution was heated to 320 °C under N_2 flow with vigorous stirring for 20 min, and cooled down to RT. The resulting $NaCeF_4$:Er/Yb@$NaCeF_4$ core/shell NCs were

precipitated by addition of 30 mL of ethanol, collected by centrifugation, washed with ethanol several times, and redispersed in cyclohexane.

Synthesis of ligand-free $NaCeF_4$:Er/Yb NCs: Ligand-free $NaCeF_4$:Er/Yb NCs were obtained by removing the surface ligands of the OA-capped counterparts through acid treatment.[1] In a typical process, 20 mg of the OA-capped $NaCeF_4$:Er/Yb NCs were dispersed in 15 mL of acidic ethanol solution (prepared by adding 112 μL of concentrated hydrochloric acid to 15 mL of absolute ethanol) and stirred for 30 min to remove the surface ligands. After that, the ligand-free $NaCeF_4$:Er/Yb NCs were collected by centrifugation, and further purified by adding an acidic ethanol solution (pH 4). The resulting products were washed with ethanol and distilled water several times, and redispersed in distilled water.

Synthesis of Lipo-modified $NaCeF_4$:Er/Yb@$NaCeF_4$:Er/Yb NCs: Because of the oleate ligands capping on the surface, the as-synthesized $NaCeF_4$:Er/Yb@$NaCeF_4$:Er/Yb NCs were hydrophobic. To render these NCs hydrophilic and biocompatible, we coated the surface of NCs with a monolayer of functional phospholipids.[2] 5 mg of OA-capped $NaCeF_4$:Er/Yb@$NaCeF_4$:Er/Yb NCs were added into a chloroform solution (3 mL) containing 20 mg of DSPE-PEG phospholipid in a round-bottom flask (5 mL), and the mixture was sonicated for 2 min. Then, the mixture was dried in a rotary evaporator under reduced pressure at 60 °C to form a lipid film on the inside wall of the flask. The lipid film was hydrated with ultrapure water (4 mL), and the NCs became soluble after vigorous sonication for 30 min. The excess lipids were removed from Lipo-NCs by ultracentrifugation (26600 g, 30 min), washing, and redispersed in distilled water.

H_2O_2 detection based on $NaCeF_4$:Er/Yb nanoprobes: Typically, 100 μL of ligand-free $NaCeF_4$:Er/Yb NCs (0.5 mg/mL) was mixed with 100 μL of deionized water containing different amounts (0-10 μM) of H_2O_2 in a 96-well microplate. After incubation at 37 °C for 2 h, the microplate was subjected to photoluminescent (PL) measurement under 980-nm excitation on a fluorescence spectrometer coupled with a multimodal microplate reader.

Uric acid detection based on $NaCeF_4$:Er/Yb nanoprobes: 100 μL of UA with different concentration was added to 100 μL of H_3BO_3-NaOH buffer solution (10 mM, pH 8.5) containing ligand-free $NaCeF_4$:Er/Yb NCs (0.5 mg/mL) and uricase (0.011U/mL) in a 96-well microplate. After incubation at 37 °C for 3 h, the microplate was subjected to PL measurement under 980-nm excitation. For

comparison, control experiments were conducted by replacing UA with other analytes (*e.g.*, metal ions, proteins, carbohydrates and amino acids) under otherwise identical conditions.

Uric acid detection in serum sample based on $NaCeF_4$:Er/Yb nanoprobes: For UA detection in human serum, a calibration curve for UA was first built based on UA depleted human serums. The collected serum samples were pretreated with Sulphur acid to deplete the protein and then NEM was added to eliminate the interference from reducing agents. The treated serum samples were then incubated with H_3BO_3-NaOH buffer solution (10 mM, pH 8.5) containing ligand-free $NaCeF_4$:Er/Yb NCs (0.5 mg/mL), uricase (0.011 U/mL), and different amounts (0-900 μM) of UA in a 96-well microplate. After incubation at 37 °C for 3 h, the microplate was subjected to spectra measurement upon 980-nm excitation. For real serum sample detection, the samples with different concentration of UA were pretreated with Sulphur acid and diluted by 100 fold with H_3BO_3-NaOH buffer solution. Three independent experiments were carried out to yield the average value and deviation.

***In vivo* bioimaging measurements:** The *in vivo* upconversion (UC) imaging experiments were performed on an IVIS Lumina II imaging system with an external 0~2 W adjustable 980-nm continuous-wave (CW) laser as the excitation source. *In vivo* NIR imaging was performed with a modified NIR vana 640 CCD camera (Princeton Instruments Inc.), with quantum efficiency of ~85% for 1530 nm. A 980-nm CW semiconductor laser was used as the excitation source, in combination with two long-pass optical filters (1000LP from Chroma Corp and 1200LP from thorlabs). A NIR-CCD Xeva-1.7-640 Xenics Co. with a band pass filter of 1538±82 nm (semrock FF01-1538/82-25) was used as the signal collector. NIR luminescence signals were analyzed with Kodak Molecular Imaging Software. For *in vivo* imaging, 1 mL of water soluble NCs (0.1 mg/mL) was imbued to the tail of a nude mouse by using gastric syringe. All the animal procedures were in agreement with the guidelines of the Institutional Animal Care and Use Committee of Fudan University and performed in accordance with institutional guidelines on animal handling.

Characterization: Powder X-ray diffraction (XRD) patterns of the samples were collected with an X-ray diffractometer (MiniFlex2, Rigaku) with Cu Kα1 radiation (λ = 0.154187 nm). Both the low- and high-resolution transmission electron microscopy (TEM) measurements were performed by using a JEOL-2010 TEM equipped with the energy dispersive X-ray (EDX) spectrum. Downshifting (DS) and UC emission spectra were carried out upon 980-nm excitation provided by a CW semiconductor laser. Thermogravimetric analysis (TGA) experiments were conducted on a Netzsch STA449C thermal analysis system under N_2 atmosphere at a rate of 10 °C/min. Zeta potential and hydrodynamic diameter

distribution of the NCs were determined by means of dynamic light scattering (DLS) measurement (Nano ZS ZEN3600, Malvern). Fourier-transform infrared (FTIR) spectra were recorded in KBr discs on a Magna 750 FTIR spectrometer. The absolute quantum yield (QY) of $NaCeF_4$:Er/Yb NCs was measured at RT by employing a barium sulfate coated integrating sphere (Edinburgh) as the sample chamber that was mounted on the FLS920 spectrometer with the entry and output port of the sphere located in 90° geometry from each other in the plane of the spectrometer. All the spectral data collected were corrected for the spectral response of both the spectrometer and the integrating sphere. Photographs of the NCs solution were taken by a Canon digital camera without using any filter. The homogeneous assays of H_2O_2 and UA were carried out on a custom-built microplate reader.

II. Supplementary Table

Table S1. Comparison of the UA levels in 24 human serum samples determined based on ligand-free $NaCeF_4$:Er/Yb nanoprobes and commercial kit (mean ± standard deviation (SD), coefficient of variation (CV), n = 3).

	Assay based on $NaCeF_4$:Er/Yb		Assay based on Commercial kit		Assay based on $NaCeF_4$:Er/Yb		Assay based on Commercial kit
No.	Mean ± SD	CV (%)	Mean ± SD	No.	Mean ± SD	CV (%)	Mean ± SD
1	278.5 ± 3.6	1.3	262.5 ± 2.5	13	139.8 ± 2.3	1.6	89.7 ± 3.1
2	423.3 ± 2.7	0.6	400.4 ± 3.1	14	296.2 ± 5.7	1.9	246.5 ± 4.1
3	612.8 ± 5.2	0.8	550.2 ± 4.9	15	510.7 ± 4.3	0.8	479.5 ± 2.2
4	71.3 ± 0.9	1.2	86.3 ± 0.7	16	371.3 ± 7.1	1.9	384.6 ± 2.2
5	189.4 ± 1.2	0.9	200.4 ± 1.2	17	502.7 ± 5.2	1.0	543.7 ± 4.2
6	360.4 ± 3.2	0.6	380.3 ± 3.0	18	700.4 ± 4.0	0.6	684.2 ± 2.5
7	221.4 ± 1.4	0.6	235.8 ± 2.2	19	278.1 ± 3.4	1.2	188.6 ± 0.9
8	356.4 ± 5.9	1.7	386.3 ± 4.2	20	391.3 ± 6.6	1.7	362.2 ± 3.6
9	564.3 ± 6.3	1.1	523.7 ± 4.4	21	612.6 ± 2.3	0.4	571.6 ± 3.0
10	115.4 ± 4.9	4.2	109.3 ± 1.2	22	179.2 ± 5.7	3.2	152.5 ± 2.9
11	247.2 ± 6.6	2.5	261.4 ± 1.5	23	254.4 ± 2.7	1.1	296.8 ± 3.5
12	409.6 ± 2.0	0.5	438.7 ± 3.6	24	521.9 ± 3.5	0.7	489.7 ± 4.3

III. Supplementary Figures

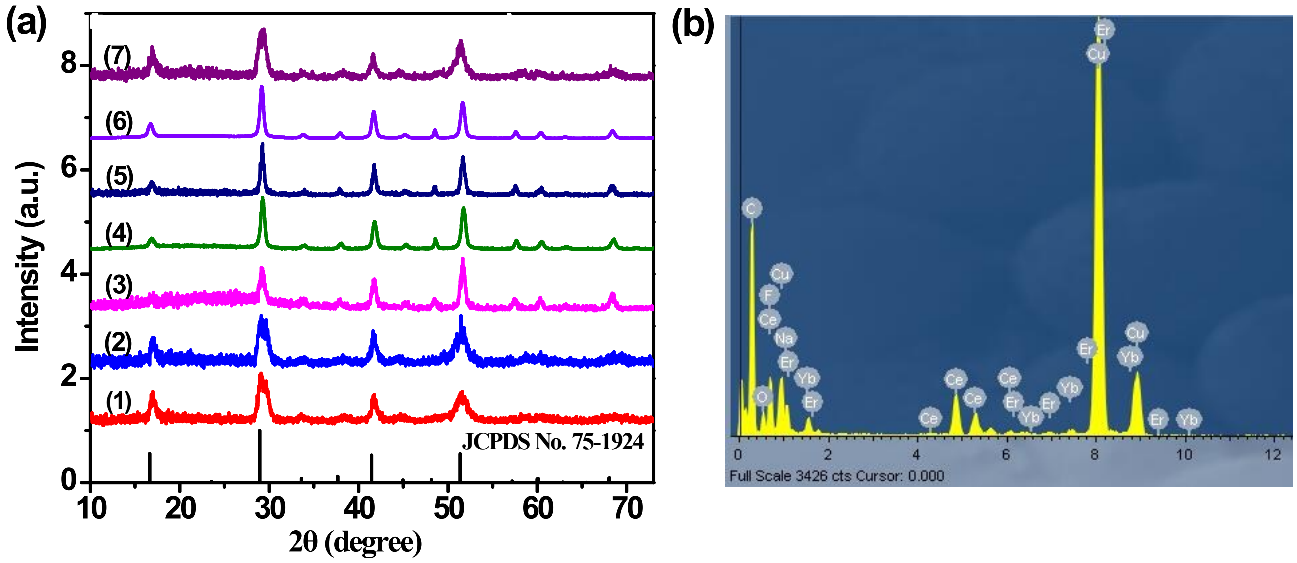

Figure S1. (a) Powder XRD patterns of $NaCeF_4$:Er/Yb NCs via high-temperature co-precipitation reaction at 320 °C for (1) 20 min, (2) 25 min, (3) 30 min, (4) 45 min, (5) 60 min, (6) 90 min, respectively. (7) is the XRD pattern of $NaCeF_4$:Er/Yb@$NaCeF_4$ core/shell NCs. All diffraction peaks of the synthesized NCs can be well matched with the standard pattern of hexagonal $NaCeF_4$ (JCPDS No. 75-1924, black lines) and no other impurity peaks were detected. (b) EDX spectroscopy analysis of $NaCeF_4$:Er/Yb NCs, which reveals the existence of Yb and Er elements in $NaCeF_4$ NCs.

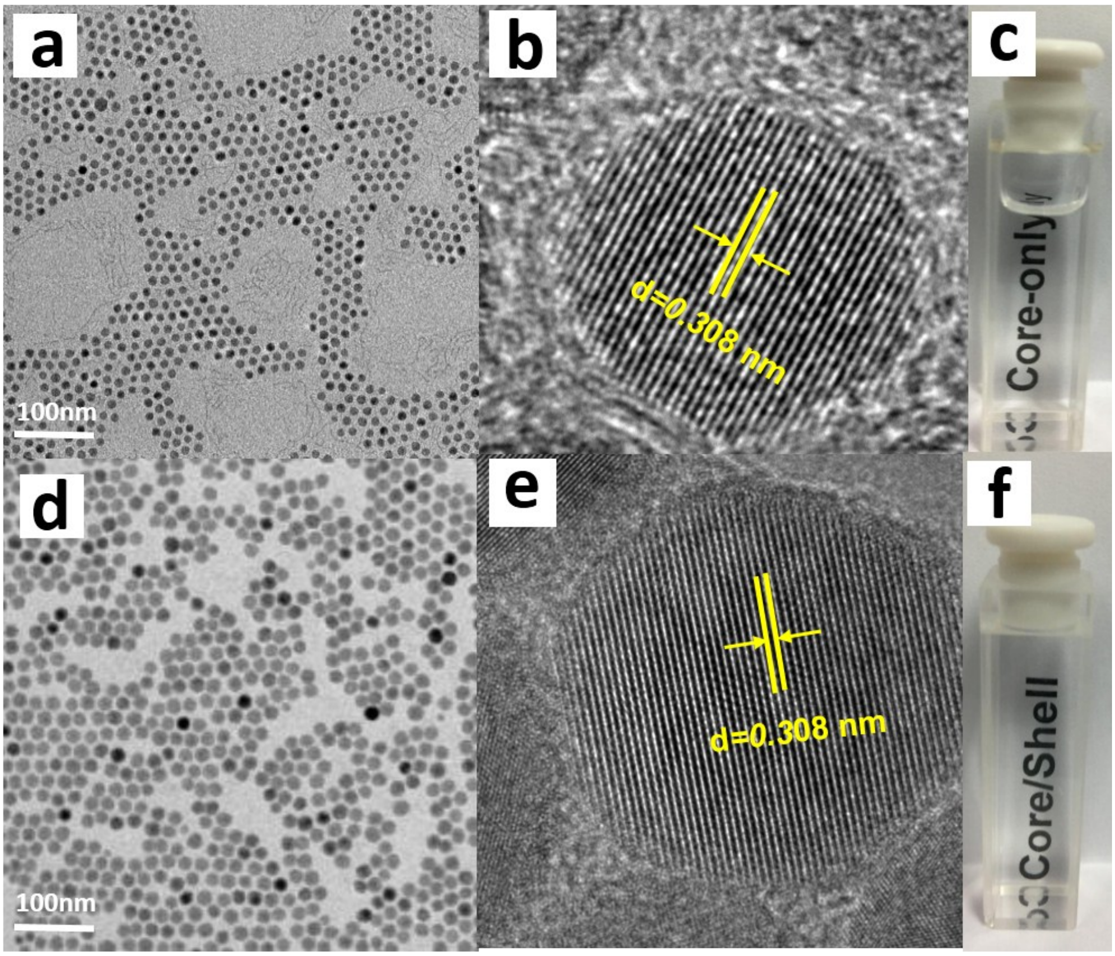

Figure S2. (a) TEM and (b) high-resolution TEM (HRTEM) images of $NaCeF_4$:Er/Yb core-only NCs. (c) Photograph of $NaCeF_4$:Er/Yb core-only NCs dispersed in cyclohexane solution. (d) TEM and (e) HRTEM images of $NaCeF_4$:Er/Yb@$NaCeF_4$ core/shell NCs. (f) Photograph of $NaCeF_4$:Er/Yb@$NaCeF_4$ core/shell NCs dispersed in cyclohexane solution. The HRTEM images for both $NaCeF_4$:Er/Yb core-only and $NaCeF_4$:Er/Yb@$NaCeF_4$ core/shell NCs show a clear observed d-spacing of 0.308 nm, which is in good agreement with the lattice spacing in $(01\bar{1}1)$ plane of hexagonal $NaCeF_4$ (JCPDS No. 75-1924), indicative of the high crystallinity of the as-prepared NCs.

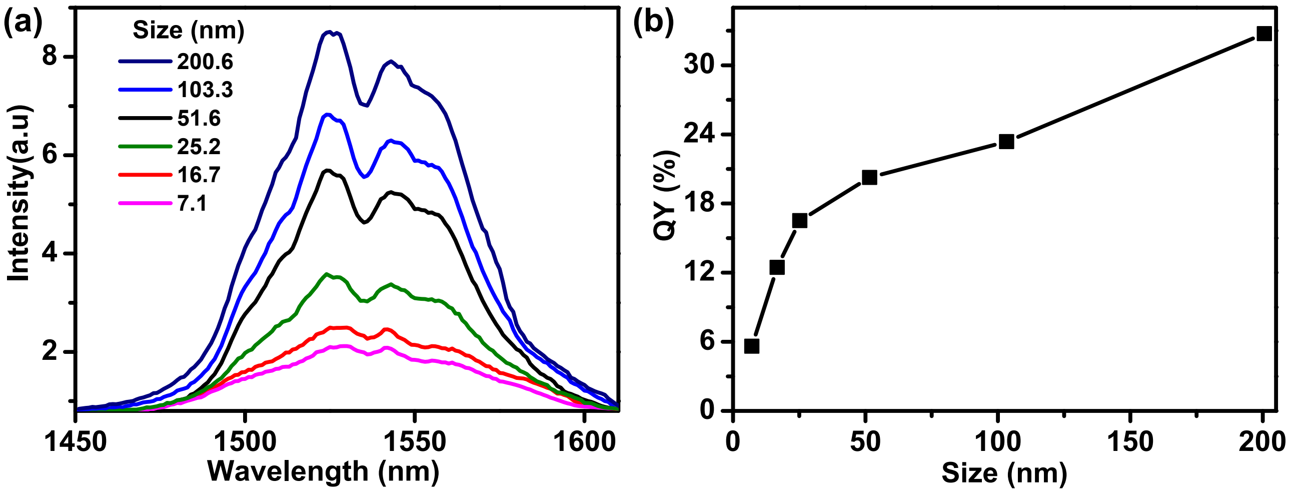

Figure S3. (a) NIR-II emission spectra of $NaCeF_4$:Er/Yb core-only NCs with different size upon excitation at 980 nm. The emission intensity of $NaCeF_4$:Er/Yb NCs increased by 4.1 times as the size increased from 7.1 to 200.6 nm. (b) Absolute NIR-II luminescent QY for $NaCeF_4$:Er/Yb NCs with different size upon excitation at 980 nm with power density of ~100 Wcm^{-2}. Specifically, the maximum NIR-II QY for the $NaCeF_4$:Er/Yb NCs (200.6 ± 16.5 nm) was determined to be 32.8%.

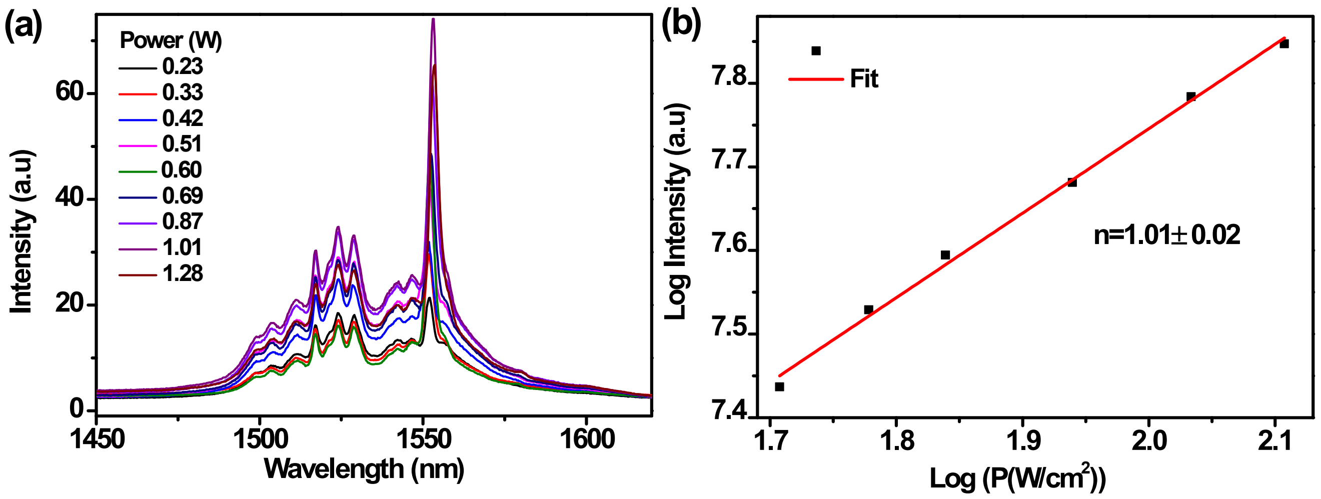

Figure S4. (a) 10 K emission spectra of $NaCeF_4$:Er/Yb NCs upon excitation at 980 nm with different power density. (b) Log–log plots of the NIR-II emission intensity versus 980-nm excitation power density for $NaCeF_4$:Er/Yb NCs. Upon excitation at 980 nm, emission intensity (I) is proportional to the nth power of P, namely, $I \propto P^n$, where n is the number of absorbed photon for one emitted photon. A plot of log I versus log P yields a straight line with slope n. The slope for $^4I_{13/2} \rightarrow {}^4I_{15/2}$ transition of Er^{3+} was determined to be 1.01 ± 0.02, which infers that the NIR-II emission occurred via one-photon process.

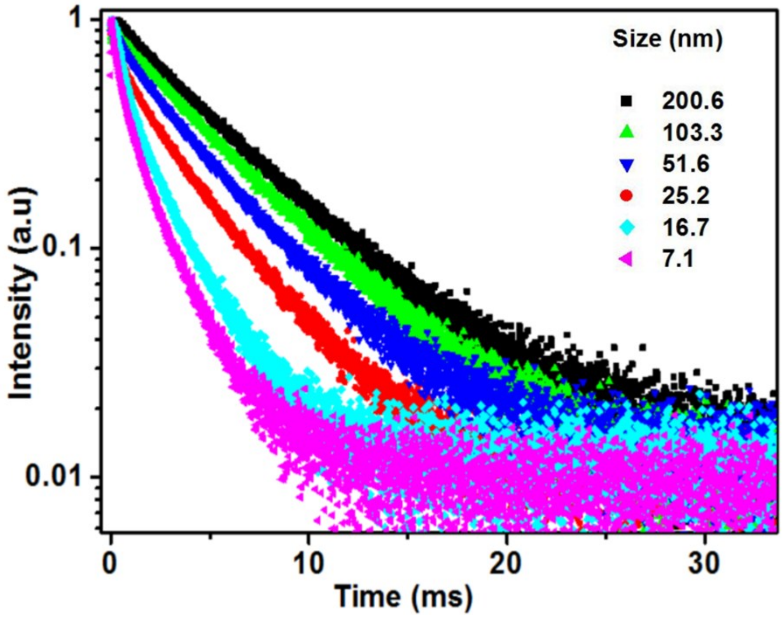

Figure S5 PL decays of the $^4I_{13/2}$ level of Er^{3+} in $NaCeF_4$:Er/Yb NCs with different size by monitoring the emission at 1530 nm, upon excitation at 980 nm. The effective lifetime τ_{eff} of Er^{3+}, defined by $(1/I_0)\int_0^\infty I(t)dt$, increased from 1.53 to 5.60 ms as the size increased from 7.1 to 200.6 nm.

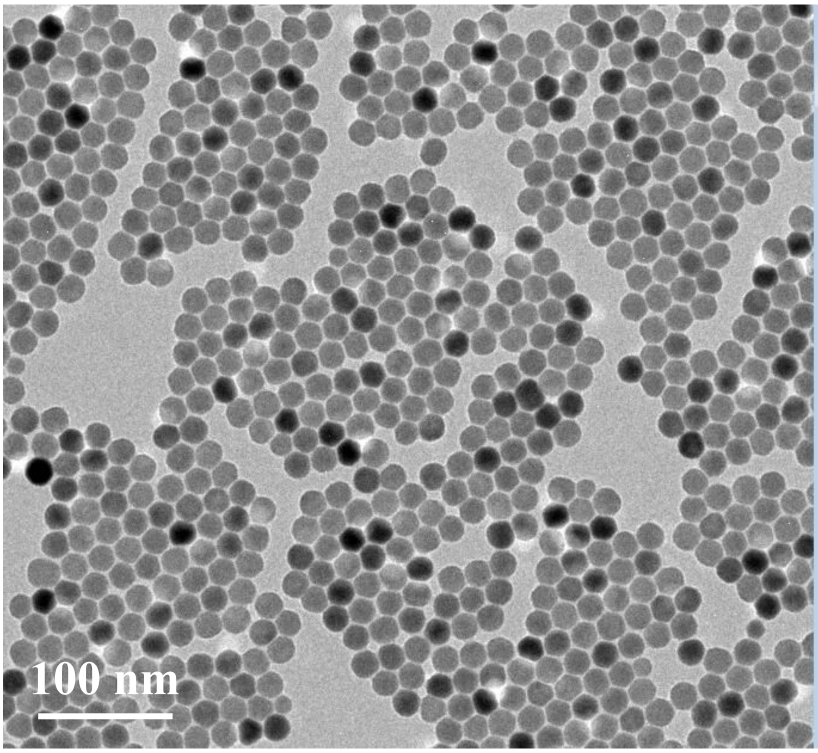

Figure S6 TEM image of $NaYF_4$:Er/Yb NCs. The size of $NaYF_4$:Er/Yb NCs was determined to be 20.1 ± 1.8 nm by randomly analyzing 200 particles. Inductively coupled plasma atomic emission spectroscopy (ICP-AES) reveals the existence of the doped Er (0.95 mol%) and Yb (20.18 mol%) ions in $NaYF_4$:Er/Yb NCs. The doping contents of Er and Yb ions in $NaYF_4$:Er/Yb NCs are essentially the same as those of $NaCeF_4$:Er/Yb NCs.

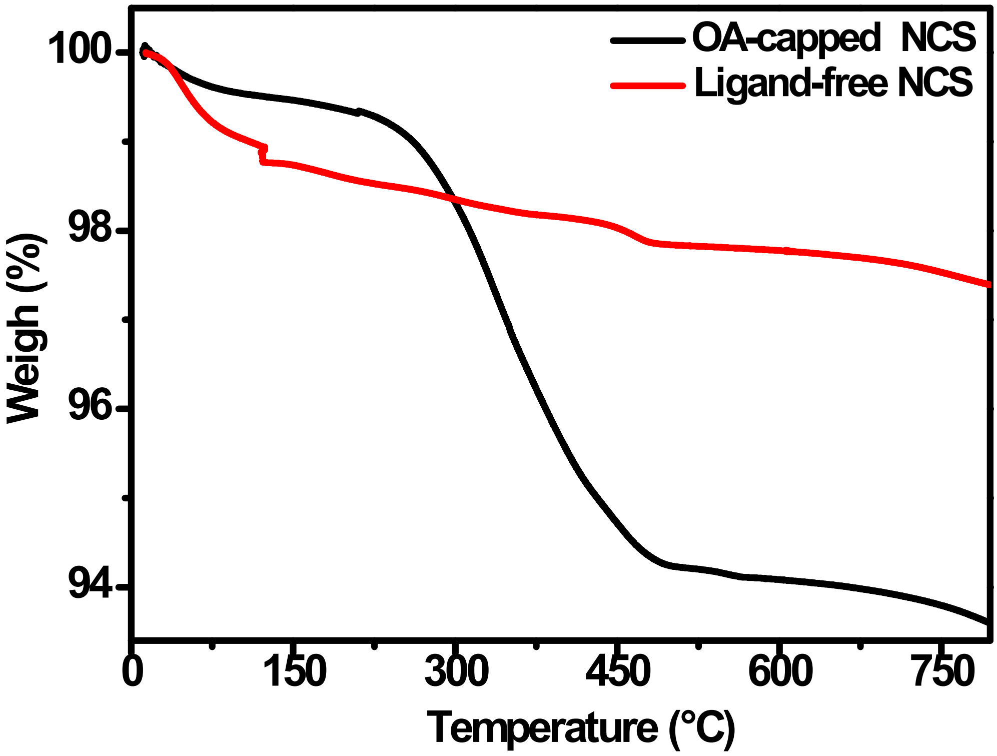

Figure S7. (a) TGA curves of the OA-capped and ligand-free $NaCeF_4$:Er/Yb NCs obtained by heating them under N_2 atmosphere in the temperature range of 20–800 °C at a rate of 10 °C/min. Different decomposition temperatures and weight losses were observed, due to the different ligands capping on the surface of NCs.

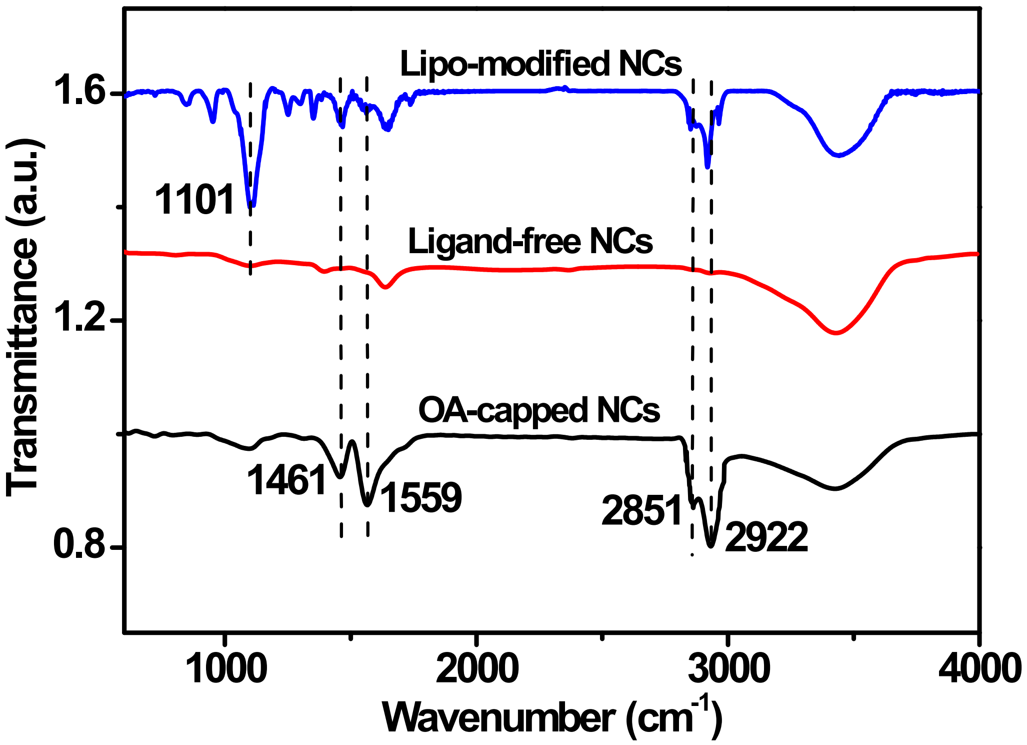

Figure S8. FTIR spectra of OA-capped, ligand-free and Lipo-modified NCs, respectively. After acid treatment of the OA-capped NCs, the original asymmetric and symmetric stretching vibrations of methylene (-CH_2-) in the long alkyl chain peaking at 2922 and 2851 cm^{-1}, and the asymmetric and symmetric stretching vibrations of the carboxylic group (-COO^{-}) peaking at 1559 and 1461 cm^{-1} disappeared in the ligand-free NCs. These features corroborate the successful removal of oleate ligands from the surface of NCs. Besides, a new peak at 1101 cm^{-1} appearing on the Lipo-NCs was ascribed to the stretching vibrations of the C−O−C bond in DSPE-PEG phospholipid, indicating that the DSPE-PEG phospholipid was successfully assembled on the OA-NCs surface.

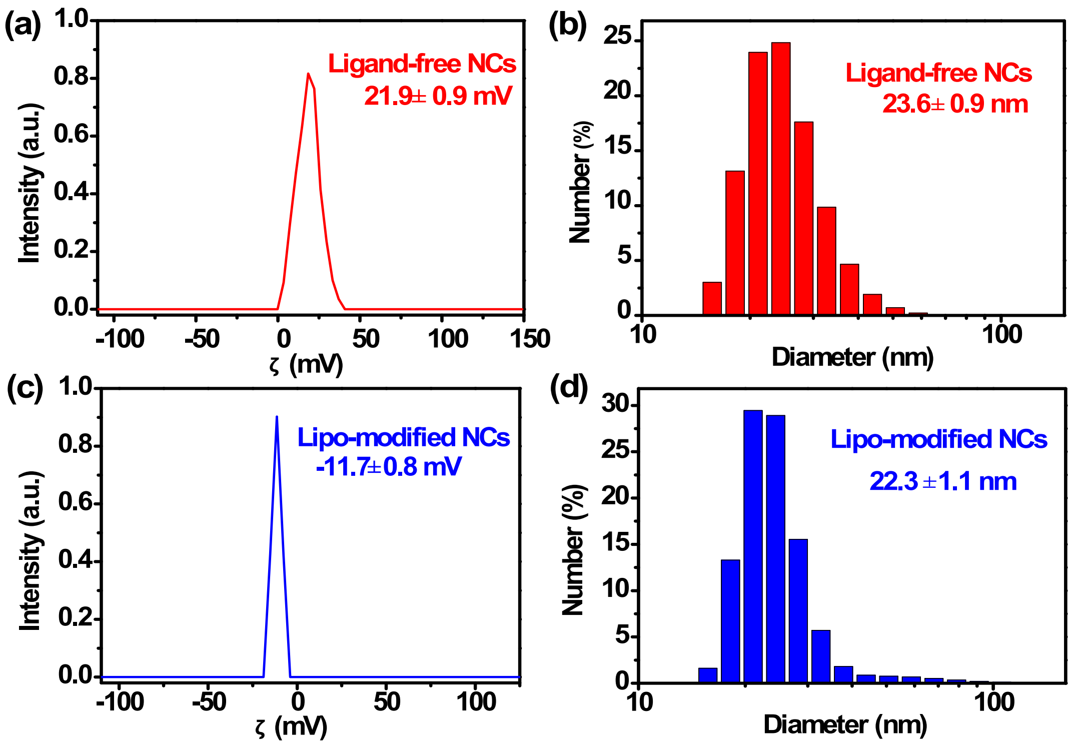

Figure S9. (a) Hydrodynamic diameter (HD) distribution and (b) ζ-potential of ligand-free $NaCeF_4$:Er/Yb NCs. (c) HD distribution and (b) ζ-potential of Lipo-modified $NaCeF_4$:Er/Yb@$NaCeF_4$ NCs. The ζ-potential for ligand-free $NaCeF_4$:Er/Yb NCs dispersed in aqueous solution (pH 6.9) was determined to be 21.9 ± 0.9 mV due to the existence of positively charged Ln^{3+} ions (*i.e.*, Er^{3+}, Yb^{3+} and Ce^{3+}) on the surface of ligand-free NCs. By contrast, the ζ-potential for Lipo-modified $NaCeF_4$:Er/Yb@$NaCeF_4$ NCs was determined to be -11.7 ± 0.8 mV, as a result of the negatively charged DSPE-PEG phospholipid bound on the NC surface. Besides, the ligand-free $NaCeF_4$:Er/Yb NCs had a HD of 23.6 ± 0.9 nm, which is similar to the size of OA-capped $NaCeF_4$:Er/Yb NCs (25.2 ± 2.7 nm). Meanwhile, the Lipo-modified $NaCeF_4$:Er/Yb@$NaCeF_4$ NCs had a HD of 22.3 ± 1.1 nm, which is a little larger than that of OA-capped $NaCeF_4$:Er/Yb@$NaCeF_4$ NCs (18.1 ± 1.9 nm) due to the coating of DSPE-PEG phospholipid on the NC surface.

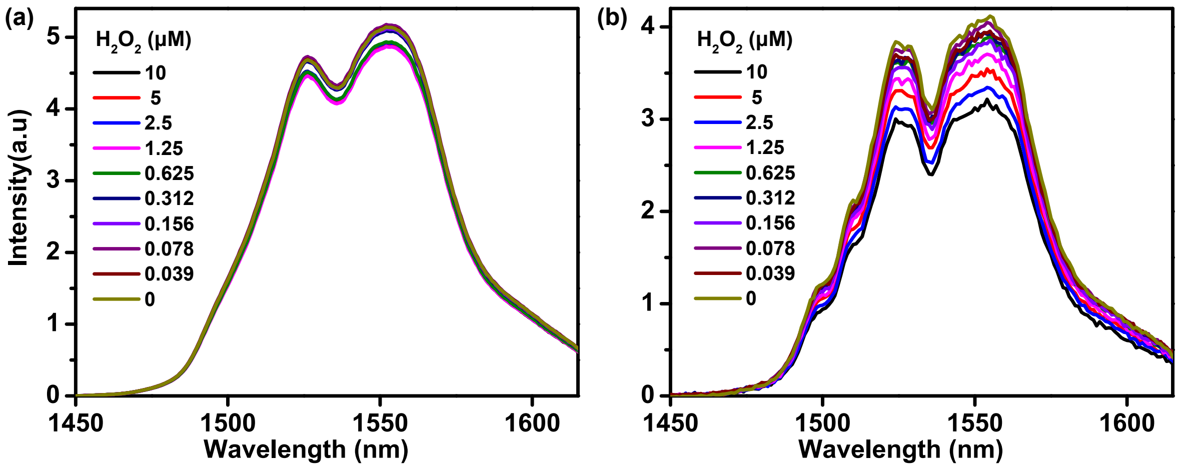

Figure S10. Detection of H_2O_2 based on ligand-free (a) $NaYF_4$:Er/Yb and (b) $NaYF_4$:Er/Yb/Ce (with Ce^{3+} content of 10 mol%) nanoprobes (0.5 mg/mL) upon excitation at 980 nm. Negligible quenching effect on the NIR-II emission of Er^{3+} was observed upon addition of H_2O_2 with different concentration (0-10 μM).

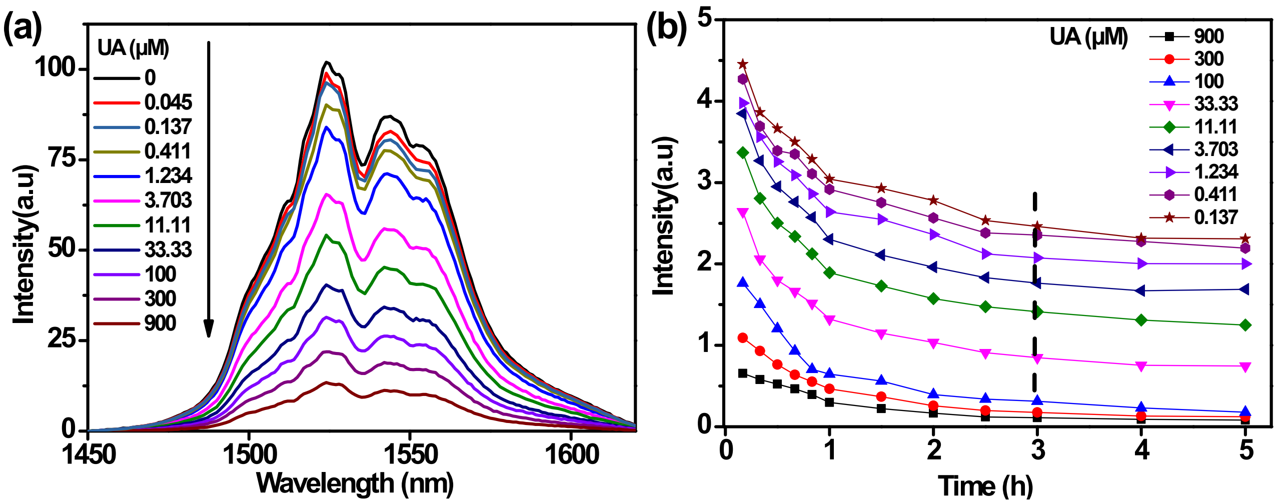

Figure S11. (a) Detection of UA based on ligand-free $NaCeF_4$:Er/Yb nanoprobes (0.5 mg/mL) upon excitation at 980 nm. In the assay system, the concentration of uricase was 0.011U/mL. The NIR-II emission intensity was gradually decreasing along with the addition of UA. (b) Time-dependent NIR-II signal of ligand-free $NaCeF_4$:Er/Yb nanoprobes upon addition of UA with different concentrations. According to the intensity of NIR-II emission of Er^{3+}, the reaction reached equilibrium after 3 h.

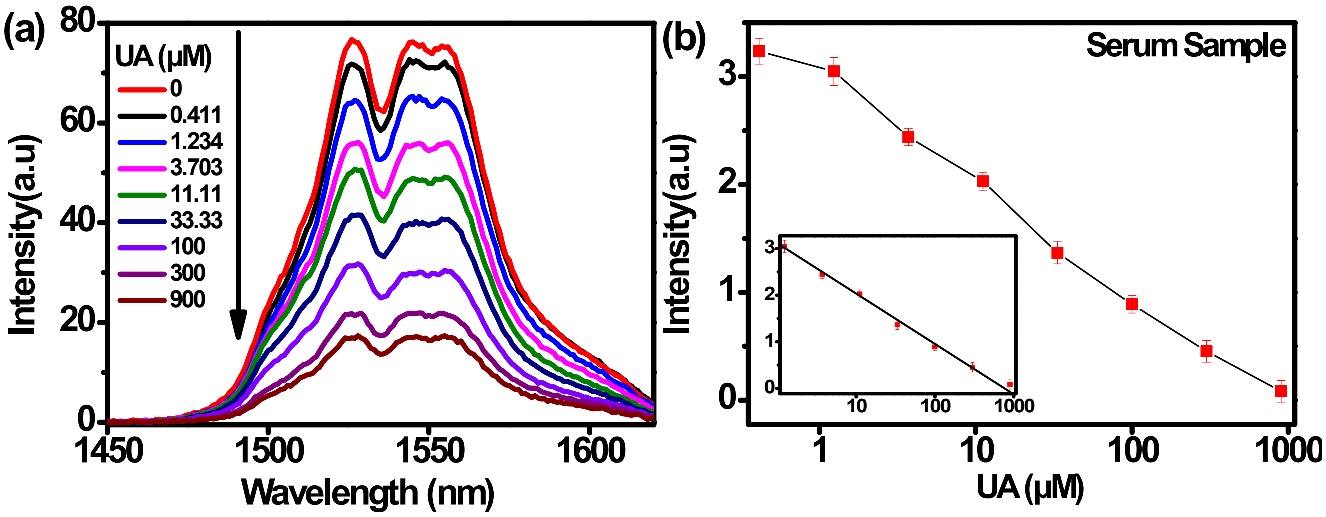

Figure S12. (a) Detection of UA in serum based on ligand-free $NaCeF_4$:Er/Yb nanoprobes (0.5 mg/mL) upon excitation at 980 nm. (b) Calibration curve for the UA assay in serum. Inset shows the linear range (1.234 – 900 μM) of the calibration curve.

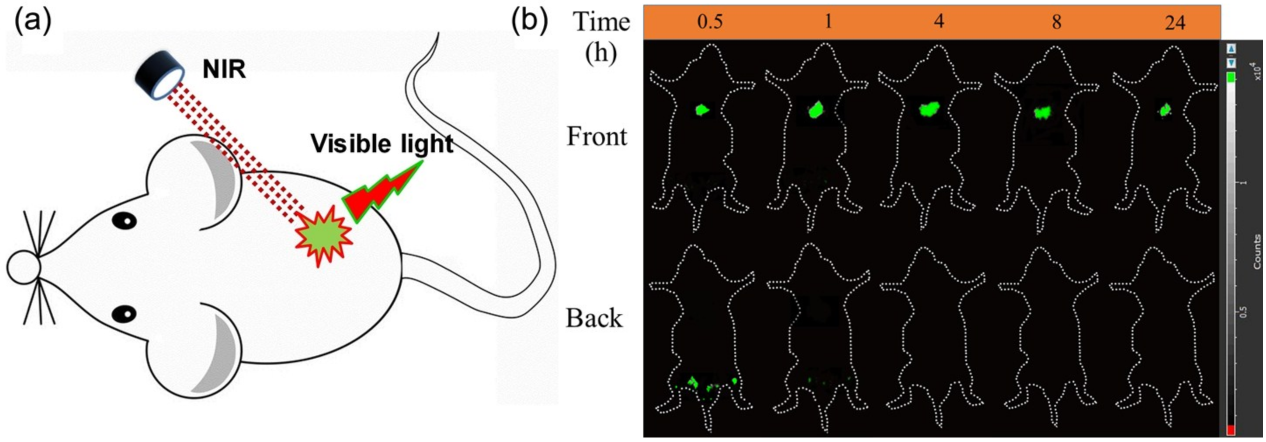

Figure S13. (a) Schematic illustration of *in vivo* imaging by utilizing the visible UC emission of $NaYF_4$:Er/Yb upon excitation at 980 nm (0.2 Wcm^{-2}). (b) Photographs for *in vivo* front and back images of nude mice after tail vein injection of Lipo-modified $NaYF_4$:Er/Yb NCs (0.1 mg/mL, 1 mL) at different times.

Reference

1. P. Huang, W. Zheng, S. Zhou, D. Tu, Z. Chen, H. Zhu, R. Li, E. Ma, M. Huang and X. Chen, *Angew. Chem. Int. Ed.*, 2014, **53**, 1252-1257.
2. Y. Cen, Y. M. Wu, X. J. Kong, S. Wu, R. Q. Yu and X. Chu, *Anal. Chem.*, 2014, **86**, 7119-7127.
